# Interpretable machine learning models using early dynamic clinical features to predict early hemodynamic stabilization after vasopressor initiation in patients with septic shock: development in MIMIC-IV and external validation in eICU

**DOI:** 10.3389/fmed.2026.1920329

**Published:** 2026-09-16

**Authors:** Shu-jiao Lu, Ze-kun Wei, Guo-chen Li, Qian-yu Bi, Le-run Zong, Li Kong, Fei-hu Zhang

**Affiliations:** 1 Shandong University of Traditional Chinese Medicine, Jinan, China; 2 Department of Emergency Center, Affiliated Hospital of Shandong University of Traditional Chinese Medicine, Jinan, China

**Keywords:** early hemodynamic stabilization, external validation, lactate clearance, machine learning, MIMIC-IV, norepinephrine-equivalent dose, septic shock, SHAP

## Abstract

**Background:**

Early hemodynamic stabilization after vasopressor initiation in septic shock is heterogeneous and difficult to predict. We aimed to develop and externally validate interpretable machine learning models for predicting early hemodynamic stabilization.

**Methods:**

This retrospective study used MIMIC-IV for model development and internal validation and eICU for external validation. Adult ICU patients with septic shock receiving vasopressors were included. Dynamic clinical features during the first 6 h after vasopressor initiation were used to predict stabilization during 6–24 h, defined by sustained mean arterial pressure (MAP) control without vasopressor escalation or increased norepinephrine-equivalent dose. Seven machine learning models were compared. Performance was assessed using discrimination, calibration, Brier score, decision curve analysis, external validation, and SHAP.

**Results:**

The MIMIC-IV and eICU cohorts included 3,445 and 453 patients, with stabilization rates of 41.77% and 32.67%, respectively. Gradient boosting machine (GBM) showed the best overall performance, with an AUC of 0.720 and Brier score of 0.209 in internal validation and an AUC of 0.730 and Brier score of 0.192 in external validation. At the training-derived threshold, external sensitivity and specificity were 0.466 and 0.797. SHAP identified early MAP stability, lactate clearance, platelet count, renal function, inflammatory burden, and vasopressor intensity as major predictors. The stabilization endpoint was associated with ICU and hospital mortality and remained robust to alternative MAP definitions. However, predictive performance was substantially driven by proximal hemodynamic features, particularly MAP trajectories, and the full GBM did not consistently outperform simpler hemodynamic benchmarks externally.

**Conclusions:**

Early dynamic clinical features provided moderate discrimination for predicting hemodynamic stabilization after vasopressor initiation, with similar external performance. The model should be interpreted as a structured risk-stratification tool rather than a measure of pharmacological vasopressor responsiveness. Prospective validation is required before clinical implementation.

## 1 Introduction

Septic shock is a life-threatening subset of sepsis characterized by profound circulatory, cellular, and metabolic abnormalities and remains associated with substantial morbidity and mortality despite advances in critical care (1, 2). Early recognition, timely antimicrobial therapy, source control, fluid resuscitation, and vasopressor support are central components of contemporary septic shock management (2). Among vasopressors, norepinephrine is recommended as the first-line agent, and an initial mean arterial pressure (MAP) target of approximately 65 mmHg is commonly used during early resuscitation (2, 3). However, patients with septic shock are clinically heterogeneous, and the same vasopressor strategy may lead to different hemodynamic responses across individuals.

Early hemodynamic stabilization is clinically important because it reflects both restoration of arterial pressure and stabilization of vasoactive drug requirements. Failure to achieve early hemodynamic stabilization may indicate persistent vasoplegia, inadequate tissue perfusion, ongoing organ dysfunction, or the need for treatment escalation. Several routinely available clinical variables may be relevant to early hemodynamic stabilization. MAP reflects macrocirculatory stability, lactate clearance is a marker of improvement in global tissue hypoperfusion, and vasopressor dose intensity can quantify the degree of circulatory support (4, 5). In addition, inflammatory burden, coagulation abnormalities, renal dysfunction, respiratory compromise, and organ support may also influence or reflect the trajectory of early shock reversal. Therefore, a prediction model that integrates these early dynamic clinical features may help identify patients who are unlikely to achieve sustained hemodynamic stabilization.

Previous studies have attempted to characterize vasopressor or vasoactive drug responsiveness in septic shock. For example, machine learning has been used to predict adjunctive vasopressin responsiveness among patients with septic shock treated with norepinephrine and vasopressin (6). More recently, the blood pressure response index was proposed as a simplified indicator of cardiovascular response to vasoactive drugs and was associated with clinical outcomes across multicenter sepsis cohorts (7). These studies support the clinical relevance of assessing hemodynamic response during septic shock. However, existing work has several limitations. First, some studies focused on response to a specific second-line vasopressor rather than a broader early hemodynamic stabilization phenotype. Second, many models relied mainly on baseline or pre-treatment variables rather than dynamic features collected during the early treatment window. Third, few studies have simultaneously incorporated MAP stability, lactate clearance, vasopressor intensity, inflammatory and coagulation markers, renal function, vital signs, and organ support into an externally validated and interpretable prediction framework.

Machine learning provides a flexible approach for capturing nonlinear relationships and interactions among clinical variables, which may be particularly relevant in septic shock because treatment response is influenced by multiple interacting physiologic processes. Nevertheless, prediction models intended for clinical use should not only demonstrate discrimination but also undergo external validation, calibration assessment, clinical utility evaluation, and interpretability analysis (8, 9). Model-interpretability methods, such as SHAP analysis, can characterize how individual predictors contribute to model outputs and improve transparency. However, such attribution methods do not establish causal effects, treatment effects, or underlying physiological mechanisms.

Therefore, this study aimed to develop and externally validate interpretable machine learning models using early dynamic clinical features to predict early hemodynamic stabilization after vasopressor initiation in patients with septic shock. We used the MIMIC-IV database for model development and internal validation and the eICU Collaborative Research Database for external validation. Early dynamic features were extracted during the first 6 h after vasopressor initiation, and early hemodynamic stabilization was assessed during the subsequent 6–24-hour window using an operational composite endpoint incorporating sustained MAP control and absence of vasopressor escalation. We further evaluated model calibration, decision-curve analysis, model interpretability, endpoint validity, alternative endpoint definitions, simple clinical baseline models, and hourly-binned MAP sensitivity analyses.

## 2 Materials and methods

### 2.1 Study design and data sources

This retrospective cohort study was designed to develop, internally validate, externally validate, and interpret machine learning models for predicting early hemodynamic stabilization after vasopressor initiation in patients with septic shock. The study was reported with reference to the TRIPOD+AI guidance for clinical prediction model studies (10).

The Medical Information Mart for Intensive Care IV database, version 3.1 (MIMIC-IV v3.1), was used for model development and internal validation, whereas the eICU Collaborative Research Database, version 2.0 (eICU-CRD v2.0), was used for external validation (11, 12). MIMIC-IV is a publicly available electronic health record database derived from patients admitted to the emergency department or intensive care units at Beth Israel Deaconess Medical Center and contains clinical data from 2008 to 2022. In contrast, eICU-CRD is a multicenter critical care database comprising ICU admissions from multiple hospitals across the United States during 2014–2015. The use of these two databases therefore provided an independent cross-database and multicenter evaluation while also representing different healthcare systems and calendar periods.

Both databases contain de-identified clinical data and are available through PhysioNet under credentialed access. The requirement for individual informed consent was waived by the original database oversight committees because the data were de-identified.

### 2.2 Study population

Adult ICU patients meeting a prespecified operational septic shock phenotype were eligible for inclusion. Because the adequacy of preceding fluid resuscitation could not be reliably reconstructed in either retrospective database, the study phenotype was based on sepsis, vasopressor treatment, and hyperlactatemia rather than claiming exact replication of the full clinical Sepsis-3 septic shock definition.

#### 2.2.1 MIMIC-IV cohort

Data extraction and cohort construction were performed using PostgreSQL version 17.2. Only the first ICU stay for each patient was retained. Sepsis was identified using the mimiciv_derived.sepsis3 derived table. Suspected infection was defined using temporally paired antimicrobial administration and microbiological culture according to the MIMIC-IV derived concept, with the earlier qualifying antimicrobial or culture timestamp used as the suspected-infection time. Sepsis-3 required a SOFA score ≥2 within 48 h before to 24 h after suspected infection. Vasopressor initiation defined T0. Baseline lactate was defined as the blood lactate measurement closest to T0 within a window from 6 h before to 1 h after vasopressor initiation; ties were resolved according to the prespecified extraction ordering. A baseline lactate concentration >2 mmol/L was required. For the primary analysis, an eligible follow-up lactate measurement meeting the prespecified timing criteria was required to calculate lactate clearance. Patients without such a follow-up measurement therefore could not contribute to the primary lactate-clearance-based cohort. This requirement was subsequently relaxed in a sensitivity analysis using baseline lactate alone.

#### 2.2.2 eICU cohort

Because a directly equivalent MIMIC-IV Sepsis-3 derived table was not available in eICU, sepsis was identified using a database-specific diagnosis-based phenotype constructed from recorded clinical diagnosis information and diagnostic coding. Septic shock diagnoses were identified from diagnosis strings explicitly indicating septic shock and corresponding diagnostic codes. The eICU cohort was subsequently restricted according to the same study-level clinical concepts used in MIMIC-IV, including adult status, first ICU stay, vasopressor treatment, baseline lactate >2 mmol/L, and availability of the required predictor and outcome windows. This approach preserved clinically comparable eligibility criteria while acknowledging differences in database structure and sepsis operationalization.

### 2.3 Landmark framework and time-window definition

A landmark prediction framework was used to preserve the temporal order between predictors and outcome assessment and to minimize information leakage. For all included patients, the index time, T0, was uniformly defined as the first documented time of vasopressor initiation after ICU admission. The first 6 h after T0 were defined as the early predictor window. During this T0–6-hour period, early dynamic clinical features were extracted, including hemodynamic measurements, lactate dynamics, vasopressor exposure, vital signs, laboratory indicators, and organ support.

The 6-hour time point after vasopressor initiation was defined as the landmark time, at which model prediction was performed. The subsequent T6–24-hour period was used for outcome assessment. This design ensured that all model predictors were available before the prediction time point and before outcome ascertainment. Because model prediction was performed at the 6-hour landmark, patients who died or left the ICU before T6 were not eligible to enter the landmark cohort. Deaths occurring after the landmark during the T6–24-hour outcome window were retained and classified as non-stabilization. Pre-landmark mortality and ICU departure were additionally quantified in separate candidate-cohort audits conducted before conditioning on survival and continued ICU stay to the 6-hour landmark.

### 2.4 Outcome definition

The primary outcome was early hemodynamic stabilization during the T6–24-hour period after vasopressor initiation. Because no universally accepted gold standard exists for early vasopressor responsiveness in retrospective ICU databases, this endpoint was defined as an operational composite measure of favorable early hemodynamic stabilization after vasopressor initiation rather than a direct biological measure of pharmacological vasopressor responsiveness. The composite endpoint was designed to capture sustained arterial pressure control without escalation of vasoactive support (2, 3).

Patients were classified as having early hemodynamic stabilization only if all of the following criteria were met during the T6–24-hour window: (1) mean MAP was ≥65 mmHg; (2) the proportion of MAP measurements ≥65 mmHg was ≥80%; (3) the number of vasopressor classes did not increase compared with the T0–6-hour window; and (4) the mean norepinephrine-equivalent dose during T6–24 h was not higher than the mean dose during T0–6 h. Patients who failed to meet any one of these criteria were classified as not having early hemodynamic stabilization. Death during the T6–24-hour outcome window was classified as non-stabilization.

The four components were selected to capture complementary aspects of short-term hemodynamic control and vasopressor trajectory, including sustained MAP control, absence of vasopressor class escalation, and absence of increase in vasopressor dose intensity. The 65 mmHg MAP threshold was selected because it is commonly used as an initial target during septic shock resuscitation and has been evaluated in previous septic shock trials and guidelines. Norepinephrine-equivalent dose was used to summarize vasopressor treatment intensity across agents; database-specific dose conversion, body-weight handling, infusion processing, and temporal aggregation are described in Section 2.5.1 and Supplementary Table S6. By separating the T0–6-hour predictor window from the T6–24-hour outcome assessment window, this landmark-based design preserved the temporal order between predictors and outcome ascertainment and reduced the risk of information leakage (6, 7).

### 2.5 Candidate predictors

Candidate predictors were prespecified according to clinical relevance, prior evidence in septic shock, and availability and harmonizability across MIMIC-IV and eICU (2, 4–7, 13). A total of 27 predictors were considered: age, sex, admission weight, diabetes, chronic obstructive pulmonary disease, congestive heart failure, chronic renal disease, chronic liver disease, malignancy, immunocompromised status, baseline lactate, 6-hour lactate clearance, baseline white blood cell count, platelet count, creatinine, blood urea nitrogen, minimum and mean MAP, maximum heart rate and respiratory rate, minimum peripheral oxygen saturation, vasopressor-class count, mean and maximum norepinephrine-equivalent dose (NEE), vasopressin use, mechanical ventilation, and renal replacement therapy.

Baseline lactate was defined as the measurement closest to T0 within 6 h before to 1 h after vasopressor initiation. Follow-up lactate was required to occur at least 2 h after the baseline measurement and no later than T0+6 h; when multiple measurements were available, the latest eligible value was used. Lactate clearance was calculated as [(baseline lactate—follow-up lactate)/baseline lactate] × 100%, with negative values retained. Baseline white blood cell count, platelet count, creatinine, and blood urea nitrogen were defined using the measurement closest to T0 within ±6 h. Hemodynamic, vital-sign, vasopressor, and organ-support variables were derived from the T0–6-hour predictor window.

Composite severity scores were used for descriptive cohort characterization but were not included as predictors because directly comparable definitions were not consistently available across the two databases and several component variables were already represented in the predictor set. They were not used as benchmark models because the benchmark analysis was designed specifically to assess incremental predictive value beyond proximal MAP and vasopressor-intensity information. Predictors were prespecified rather than selected using data-driven feature-selection procedures to prioritize clinical interpretability and cross-database transportability.

#### 2.5.1 Vasopressor exposure and norepinephrine-equivalent dose calculation

Vasopressor exposure was derived from the mimiciv_icu.inputevents table in MIMIC-IV and the infusiondrug table in eICU. Norepinephrine, epinephrine, dopamine, phenylephrine, and vasopressin were harmonized to a norepinephrine-equivalent dose (NEE, μg/kg/min) using the prespecified conversion implemented in the extraction pipeline: NEE = norepinephrine + epinephrine + 0.01 × dopamine + 0.10 × phenylephrine + 2.5 × vasopressin (U/min) (5). Rates recorded in μg/kg/min were used directly before agent-specific conversion. For norepinephrine, epinephrine, phenylephrine, and dopamine recorded in μg/min, the infusion rate was divided by body weight before conversion. Vasopressin recorded in U/h was first converted to U/min. Patient-specific infusion weight was used when available; otherwise, admission weight was used, and weights outside 30–250 kg were not used for non-weight-based dose conversion. Records with nonpositive rates or unrecognized dose units did not contribute to NEE.

In MIMIC-IV, only positive-rate infusion records were included. When an infusion end time was missing, a 1-min duration from the recorded start time was assigned. Concurrent vasopressor components were summed over overlapping infusion intervals, whereas intervals without an active vasopressor contributed an NEE of zero. Accordingly, mean NEE was calculated as a time-weighted average over the full elapsed window (360 min for T0–6 h and 1,080 min for T6–24 h), rather than over active-infusion time only; maximum NEE represented the maximum interval-level total. Dose-only records without a positive infusion rate were not included, and temporary gaps between documented infusions contributed zero.

In eICU, drug names, dose units, and body weight were harmonized using the available infusiondrug records. Because directly comparable continuous infusion end times were not available, NEE was calculated at recorded infusion offsets: components documented at the same offset were summed, and mean and maximum NEE were summarized across valid recorded offsets within each analysis window. Unobserved infusion durations and temporary interruptions were not imputed. At least one valid NEE record in both the T0–6-h and T6–24-h windows was required for NEE-based endpoint classification. Thus, eICU NEE summaries were record-based rather than elapsed-time-weighted, and this database-specific difference was considered when interpreting external transportability. Detailed conversion and processing rules are provided in Supplementary Table S6.

### 2.6 Data preprocessing and missing data handling

Baseline characteristics and predictor missingness were summarized using original, non-imputed data. The primary cohort required an eligible repeat lactate measurement for calculation of lactate clearance and complete baseline white blood cell and platelet measurements. Remaining missing predictor values were handled within the modeling preprocessing workflow.

All preprocessing was fitted exclusively in the MIMIC-IV training set and subsequently applied unchanged to the held-out MIMIC-IV model-comparison set and eICU cohort. Missing continuous and categorical predictors were imputed using training-set medians and modes, respectively; categorical variables were dummy encoded, zero-variance terms were removed, and numeric variables were standardized when required. Unseen validation-set factor levels were handled using the locked training-set preprocessing recipe.

### 2.7 Model development in the MIMIC-IV cohort

The MIMIC-IV cohort was randomly divided into a training set (70%) and a held-out model-comparison set (30%) using stratified sampling according to early hemodynamic stabilization status, with a fixed random seed of 20260616. All preprocessing procedures, including imputation, dummy-variable encoding, removal of zero-variance predictors, and normalization when required by the modeling workflow, were fitted exclusively within the training set. After dummy encoding of categorical variables and removal of zero-variance terms, the prespecified clinical predictors yielded 38 encoded model features. The MIMIC-IV training set included 2,413 patients, corresponding to approximately 64 training observations per encoded feature. All preprocessing parameters were estimated exclusively from the MIMIC-IV training set and subsequently applied unchanged to the held-out MIMIC-IV and eICU datasets.

Seven candidate algorithms were evaluated: logistic regression, elastic net, random forest, radial-kernel support vector machine, k-nearest neighbors, gradient boosting machine (GBM), and XGBoost. Hyperparameters were optimized within the training set using five-fold cross-validation, with ROC-AUC as the optimization metric. No oversampling, undersampling, synthetic sampling, or class weighting was applied. The complete hyperparameter search spaces are provided in Supplementary Table S1.

For GBM, the tuning grid included 100, 300, and 500 trees; interaction depths of 1, 3, and 5; shrinkage values of 0.01 and 0.05; and minimum terminal-node sizes of 10 and 20 observations. The final selected GBM hyperparameters were 300 trees, interaction depth of 1, shrinkage of 0.05, and minimum node size of 10.

After hyperparameter optimization, candidate models were evaluated in the held-out 30% MIMIC-IV dataset for comparative purposes. Given the broadly comparable performance of the leading candidate algorithms, GBM was carried forward as a representative nonlinear model for detailed interpretation and external validation rather than on the basis of demonstrated superiority over the other leading algorithms. Because this held-out dataset contributed to comparative algorithm selection, its results were interpreted as model-comparison estimates rather than completely independent final performance estimates of the selected GBM model. The GBM model and preprocessing specification were subsequently locked before application to the independent eICU cohort.

### 2.8 Model evaluation and classification threshold

Performance in the held-out MIMIC-IV model-comparison set was evaluated using ROC-AUC, PR-AUC, Brier score, joint calibration intercept, calibration slope, accuracy, sensitivity, specificity, positive predictive value, negative predictive value, F1 score, and decision-curve analysis (14). ROC-AUC was reported with 95% confidence intervals for all candidate models. For the primary GBM model, 95% confidence intervals for classification metrics, Brier score, calibration-in-the-large, and calibration slope were additionally estimated using 2,000 bootstrap resamples and are reported in Supplementary Table S2. Calibration-in-the-large was estimated separately from the jointly estimated calibration intercept and was used to assess systematic over- or underprediction of the primary GBM model.

For the primary GBM model, the classification threshold was determined exclusively from cross-validated predictions generated within the MIMIC-IV training set at the optimal hyperparameter configuration. The threshold maximizing the Youden index was selected and subsequently applied unchanged to the held-out MIMIC-IV dataset and the eICU external-validation cohort. Neither the held-out MIMIC-IV dataset nor the eICU cohort was used to determine the GBM classification threshold.

### 2.9 External validation in the eICU cohort

External validation was performed in the independent eICU cohort. The preprocessing recipe fitted in the MIMIC-IV training set was directly applied to the eICU data. No model retraining, recalibration, variable re-selection, hyperparameter tuning, or classification-threshold optimization was performed in the eICU cohort before external validation. The size of the external-validation cohort was determined by the number of patients meeting the prespecified eligibility criteria rather than by a prospective sample-size target. Because the available validation sample was fixed, the precision of external performance estimates was characterized using 95% confidence intervals rather than by a retrospective power calculation. The same performance metrics used in the held-out MIMIC-IV model-comparison set were calculated in the eICU cohort, including ROC-AUC, PR-AUC, Brier score, calibration-in-the-large, calibration slope, accuracy, sensitivity, specificity, positive predictive value, negative predictive value, *F*1 score, and decision-curve analysis.

For the primary GBM model, the classification threshold derived from cross-validated predictions within the MIMIC-IV training set was applied unchanged to the eICU cohort. For the primary GBM, 95% confidence intervals for accuracy, sensitivity, specificity, positive predictive value, negative predictive value, and *F*1 score were estimated using 2,000 bootstrap resamples (Supplementary Table S2).

External calibration was further assessed using calibration-in-the-large (CITL), calibration slope, the Brier score, the integrated calibration index (ICI), and a flexible calibration curve. Ninety-five percent confidence intervals for CITL, calibration slope, Brier score, and ICI were estimated using 2,000 bootstrap resamples. The observed outcome prevalence was compared with the mean predicted probability to characterize systematic over- or underprediction. In addition, a *post-hoc* secondary logistic recalibration analysis was performed in the eICU cohort by regressing the observed outcome on the logit of the original GBM-predicted probability. Recalibrated performance was reported separately from the prespecified external-validation results and was not considered an independent validation analysis.

The distribution of the external-validation cohort across participating eICU hospitals was additionally examined. Because most hospitals contributed only a small number of eligible patients, formal hospital-level internal-external cross-validation was considered statistically unreliable. Exploratory site-specific performance was therefore summarized only for hospitals contributing at least 20 eligible patients and at least five patients in each outcome category. Exploratory subgroup performance of the locked GBM model was also evaluated according to age (<65 vs. ≥65 years), sex, ICU type, mechanical ventilation status, infection source, and baseline shock severity defined by lactate concentration (2–<4 vs. ≥ 4 mmol/L). No model retraining, updating, or subgroup-specific threshold optimization was performed. AUC with 95% confidence intervals and Brier scores were reported where subgroup sample size permitted; AUC was not estimated for subgroups with fewer than 20 patients or fewer than five patients in either outcome category.

### 2.10 Clinical benchmark and predictor–outcome circularity analysis

To quantify the extent to which predictive performance reflected continuation of the early hemodynamic trajectory, the full GBM model was compared with several prespecified simple clinical benchmark models. These included logistic regression models using mean MAP alone, minimum MAP alone, mean norepinephrine-equivalent dose (NEE) alone, and a parsimonious MAP–NEE model including mean MAP, minimum MAP, mean NEE, and maximum NEE during T0–6 h. In addition, a reduced GBM model was developed after excluding five proximal hemodynamic and vasopressor-related predictors: minimum MAP, mean MAP, vasopressor class count, mean NEE, and maximum NEE during T0–6 h. The benchmark and reduced models were developed using the same MIMIC-IV training/testing partition and preprocessing framework as the primary analysis and were subsequently applied to the eICU cohort without model updating. Model performance was compared using ROC-AUC, PR-AUC, and Brier score. Paired differences in AUC were assessed using DeLong tests, and 95% confidence intervals for differences in AUC and Brier score were estimated using 2,000 paired bootstrap resamples (15).

For direct comparison of clinical utility, decision-curve analysis was additionally performed using failure to achieve early hemodynamic stabilization as the clinically actionable event. The predicted risk of non-stabilization was calculated as one minus the predicted probability of stabilization, without retraining, updating, or recalibrating the models. The full GBM was compared with the full logistic regression model and the parsimonious MAP–NEE model. A predicted risk exceeding a given threshold was interpreted as prompting closer hemodynamic reassessment rather than automatic vasopressor escalation or treatment modification. Because no validated clinical action threshold exists for this prediction task, threshold probabilities of 0.20, 0.30, and 0.40 were considered illustrative rather than prescriptive. Absolute net benefit was reported at these thresholds, and paired differences in net benefit between the GBM and comparator models were estimated using 2,000 bootstrap resamples.

### 2.11 Model interpretability

Model interpretability was assessed for the primary GBM model using SHAP analysis. SHAP is an additive feature-attribution method based on Shapley values and was used here to characterize the contribution of individual predictors to model output rather than to infer causal or mechanistic effects (16). SHAP values for the fitted GBM were estimated using an approximate Kernel SHAP approach implemented with the kernelshap package (exact = FALSE) and visualized using shapviz. Using a fixed random seed of 20260616, 100 observations randomly sampled from the MIMIC-IV training set were used as the background reference dataset, and 300 observations randomly sampled from the held-out MIMIC-IV model-comparison set were used as the explanation dataset. Positive SHAP values indicated contributions toward a higher predicted probability of early hemodynamic stabilization, whereas negative values indicated contributions toward a lower predicted probability.

SHAP summary plots, conventional variable-importance plots, SHAP dependence plots, and partial-dependence plots were used as complementary descriptive views of model behavior. Because SHAP attribution can be distributed among correlated predictors, individual feature rankings were interpreted cautiously and were not considered estimates of independent predictor effects or evidence of biological mechanisms.

### 2.12 Sensitivity analyses

Several sensitivity analyses were performed to examine the robustness of the primary findings and address potential concerns related to predictor–outcome proximity, endpoint definition, measurement density, cohort selection, and missing data. Unless otherwise specified, sensitivity models were rebuilt using the same MIMIC-IV model-development framework described for the primary analysis, including the original training/testing strategy and training-set-based preprocessing.

First, to assess whether predictive performance was disproportionately driven by proximal vasopressor-intensity measures, two reduced-predictor analyses were performed. The first excluded mean and maximum norepinephrine-equivalent dose (NEE) during T0–6 h, and the second additionally excluded vasopressor-class count. The resulting models were evaluated internally and subsequently applied to eICU without model updating (Supplementary Table S18).

Second, the operational composite endpoint was examined through complementary validity and alternative-definition analyses. The distributions of individual endpoint components, reasons for non-stabilization, agreement among components, and associations of the composite endpoint with ICU and hospital outcomes were evaluated (Supplementary Tables S7–S10). Alternative endpoint analyses used MAP control alone, no increase in vasopressor classes, no increase in mean NEE, and a ≥20% reduction in NEE. For each alternative endpoint, the GBM was rebuilt and evaluated within the MIMIC-IV development framework; these analyses were restricted to MIMIC-IV, with eICU reserved for external validation of the prespecified primary composite endpoint (Supplementary Table S11).

Third, sensitivity to MAP measurement frequency was assessed using hourly-binned MAP values during T6–24 h. Multiple measurements within each hour were summarized by the median, and MAP control required a median MAP ≥65 mmHg in at least 80% of observed hourly intervals, with ≥12 observed intervals required. A more conservative analysis used all 18 possible hourly intervals as the denominator and treated unobserved intervals as not meeting the MAP target. Agreement with the primary endpoint was assessed using percentage agreement, reclassification rate, and Cohen's *κ*; prediction models were not refitted for this analysis (Supplementary Table S12).

Fourth, competing events and alternative outcome-classification assumptions around the landmark were examined. Death before T6, death during T6–24 h, and ICU or hospital discharge before T0+24 h were summarized. Death during the outcome window was classified as non-stabilization according to the primary definition. In MIMIC-IV, vasopressor discontinuation before T0+24 h was additionally identified from continuous infusion records; a comparable analysis was not undertaken in eICU because directly comparable infusion end times were unavailable (Supplementary Table S13). To assess possible misclassification of successful early treatment withdrawal, patients with documented vasopressor discontinuation before T0+24 h without subsequent re-initiation or death were alternatively classified as stabilized and the GBM was rebuilt. Outcome-window deaths were also explicitly forced to non-stabilization as a consistency check (Supplementary Table S19).

Finally, three analyses addressed potential cohort-selection and missing-data mechanisms. First, the MIMIC-IV cohort was reconstructed without excluding patients solely because of missing baseline white blood cell or platelet measurements, with these values handled through the established preprocessing procedure. Second, missingness indicators were added for candidate predictors with missing observations to assess whether measurement availability itself contributed materially to prediction. Third, because repeat lactate testing could introduce care-intensity-related selection, the cohort was expanded without requiring a follow-up lactate measurement; lactate clearance was removed from the predictor set and baseline lactate was retained as the sole lactate-related predictor. Repeat-lactate availability was summarized descriptively but was not included as a predictor (Supplementary Tables S19 and S20).

### 2.13 Statistical analysis

Continuous variables were summarized as medians with interquartile ranges, and categorical variables were summarized as counts with percentages. Baseline characteristics were compared between patients with and without early hemodynamic stabilization using the Wilcoxon rank-sum test for continuous variables and the chi-square test or Fisher's exact test for categorical variables, as appropriate. A two-sided *P*-value <0.05 was considered statistically significant. All analyses were performed using R software (version 4.5.0; R Foundation for Statistical Computing, Vienna, Austria).

## 3 Results

### 3.1 Study cohort and baseline characteristics

The overall study workflow and landmark prediction framework are shown in Figure 1. In the MIMIC-IV database, 94,458 ICU stays were initially screened. After restricting the cohort to adult first ICU stays, patients with sepsis and vasopressor therapy, septic shock eligibility, and sufficient early clinical data to define the predictor and outcome assessment windows, 3,445 patients were included in the final MIMIC-IV analytic cohort. The eICU cohort was used for external validation. Among 200,859 ICU stays initially screened, 453 patients met the final eligibility criteria and were included in the external validation cohort (Figure 1A). Early dynamic clinical features were extracted during the first 6 h after vasopressor initiation, and early hemodynamic stabilization was assessed during the subsequent 6–24-hour window after the 6-hour landmark (Figure 1B). In the MIMIC-IV analytic cohort, 2,006 patients did not achieve early hemodynamic stabilization and 1,439 patients achieved early hemodynamic stabilization. The baseline characteristics of the MIMIC-IV cohort are summarized in Table 1. Overall, the median age was 67 years, and 60.8% of patients were male. Patients who achieved early hemodynamic stabilization were younger than those without stabilization and had a slightly higher proportion of male patients. Several comorbidities, including COPD, congestive heart failure, chronic renal disease, chronic liver disease, and malignancy, were more common among patients without early hemodynamic stabilization (Table 1). Infection-related characteristics also differed between groups. Abdominal infection and bloodstream infection were more frequent in patients without early hemodynamic stabilization. Compared with patients who achieved early hemodynamic stabilization, patients without early hemodynamic stabilization had higher baseline and follow-up lactate concentrations, lower lactate clearance during the first 6 h, worse renal function, and more severe acid-base disturbance. They also showed more pronounced hemodynamic instability, including lower minimum and mean MAP, higher heart rate and respiratory rate, lower SpO_2_, greater vasopressor exposure, and higher norepinephrine-equivalent doses. Severity scores, including Acute Physiology Score III, SAPS II, and OASIS, were also higher among patients without early hemodynamic stabilization.

**Figure 1:**
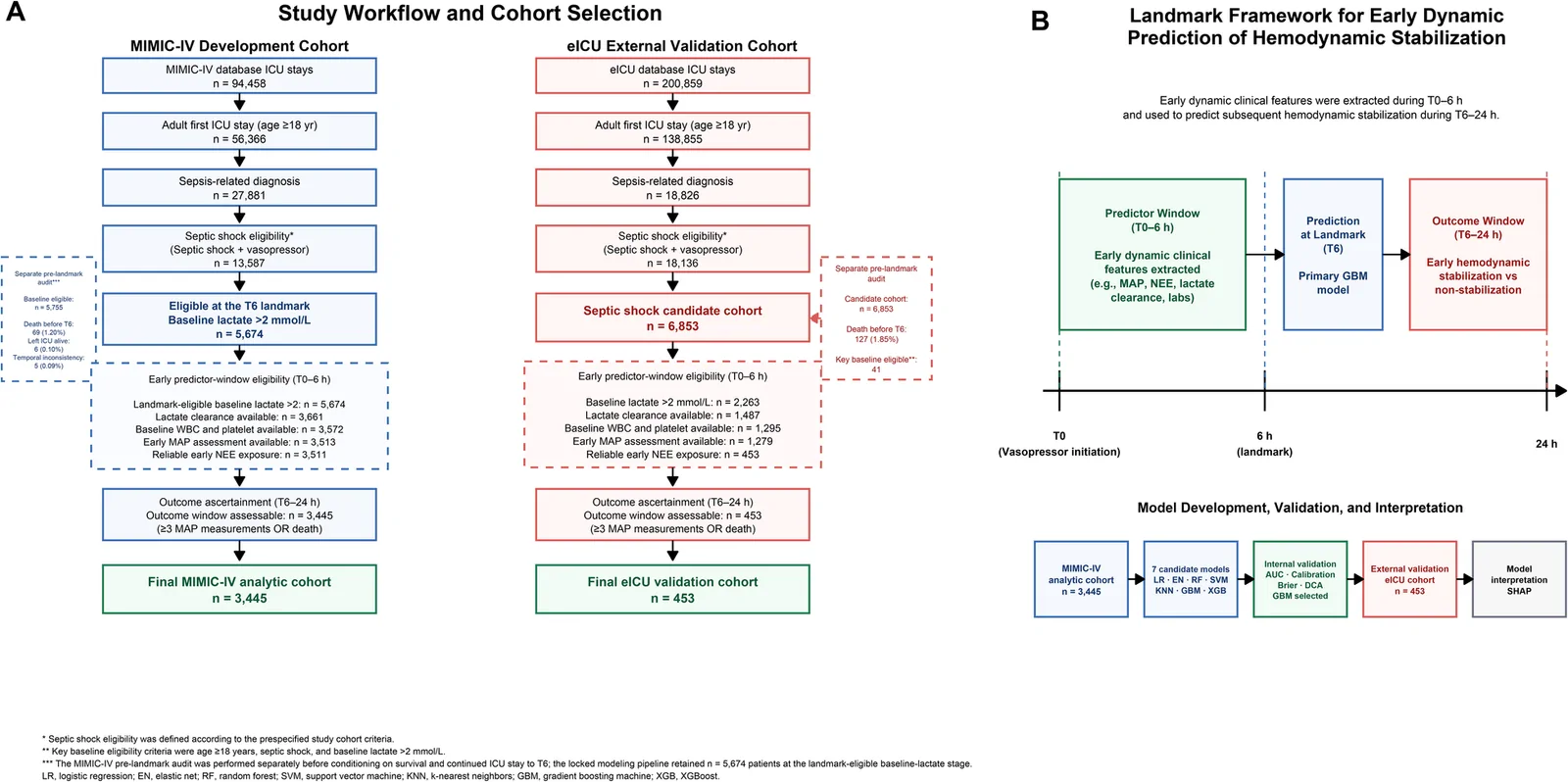
Study workflow and landmark prediction framework. **(A)** Cohort selection and model development/validation workflow in MIMIC-IV and eICU. Pre-landmark competing events were additionally assessed in separate candidate-cohort audits performed before application of the 6-hour landmark requirement. In MIMIC-IV, among 5,755 patients meeting the key baseline clinical eligibility criteria, 69 (1.20%) died before T6 and 6 (0.10%) left the ICU alive before T6; 5 additional records (0.09%) had temporally inconsistent death timestamps. In eICU, 127 of 6,853 pre-landmark candidates died before T6, including 41 patients who met the key baseline eligibility criteria of age ≥18 years, septic shock, and baseline lactate >2 mmol/L. **(B)** Landmark framework for early dynamic prediction of hemodynamic stabilization. T0 was defined as vasopressor initiation. Predictor features were extracted during T0–6 h, prediction was performed at the 6-hour landmark (T6), and early hemodynamic stabilization was assessed during the subsequent T6–24-h outcome window. MIMIC-IV was used for model development and internal validation, whereas eICU was used for external validation.

**Table 1:** Baseline characteristics of the MIMIC-IV analytic cohort stratified by early hemodynamic stabilization.

Characteristic	Overall (*N* = 3445)	Non-stabilization (*N* = 2006)	Stabilization (*N* = 1439)	*P* value
Demographics and ICU characteristics				
Age, years	67.00 [56.00, 77.00]	69.00 [58.00, 78.00]	64.00 [54.00, 74.00]	**<0** **.** **001**
Male sex, *n* (%)	2095 (60.8)	1189 (59.3)	906 (63.0)	**0** **.** **029**
Ethnicity, *n* (%)				**0** **.** **449**
White	2189 (63.5)	1294 (64.5)	895 (62.2)	
Black	241 (7.0)	131 (6.5)	110 (7.6)	
Asian	113 (3.3)	64 (3.2)	49 (3.4)	
Hispanic/Latino	119 (3.5)	63 (3.1)	56 (3.9)	
Other/unknown	783 (22.7)	454 (22.6)	329 (22.9)	
Admission weight, kg	80.60 [68.50, 95.10]	80.00 [68.00, 94.90]	81.30 [69.60, 96.50]	0.070
ICU type, *n* (%)				**<0** **.** **001**
Cardiovascular ICU	1701 (49.4)	944 (47.1)	757 (52.6)	
Medical or mixed ICU	962 (27.9)	635 (31.7)	327 (22.7)	
Surgical/trauma ICU	772 (22.4)	422 (21.0)	350 (24.3)	
Other ICU	10 (0.3)	5 (0.2)	5 (0.3)	
Comorbidities				
Hypertension, *n* (%)	2272 (66.0)	1323 (66.0)	949 (65.9)	0.998
Diabetes, *n* (%)	1073 (31.1)	639 (31.9)	434 (30.2)	0.289
COPD, *n* (%)	436 (12.7)	277 (13.8)	159 (11.0)	**0** **.** **016**
Congestive heart failure, *n* (%)	1026 (29.8)	632 (31.5)	394 (27.4)	**0** **.** **009**
Coronary artery disease, *n* (%)	1429 (41.5)	825 (41.1)	604 (42.0)	0.619
Myocardial infarction, *n* (%)	781 (22.7)	455 (22.7)	326 (22.7)	0.985
Chronic renal disease, *n* (%)	703 (20.4)	449 (22.4)	254 (17.7)	**<0** **.** **001**
Chronic liver disease, *n* (%)	722 (21.0)	458 (22.8)	264 (18.3)	**0** **.** **001**
Malignancy, *n* (%)	510 (14.8)	328 (16.4)	182 (12.6)	**0** **.** **003**
Immunocompromised status, *n* (%)	63 (1.8)	36 (1.8)	27 (1.9)	0.860
Cerebrovascular disease, *n* (%)	280 (8.1)	157 (7.8)	123 (8.5)	0.445
Peripheral vascular disease, *n* (%)	190 (5.5)	122 (6.1)	68 (4.7)	0.085
Infection sites				
Respiratory infection, *n* (%)	570 (16.5)	345 (17.2)	225 (15.6)	0.224
Urinary infection, *n* (%)	464 (13.5)	280 (14.0)	184 (12.8)	0.321
Abdominal infection, *n* (%)	440 (12.8)	290 (14.5)	150 (10.4)	**<0** **.** **001**
Bloodstream infection, *n* (%)	1203 (34.9)	807 (40.2)	396 (27.5)	**<0** **.** **001**
Skin and soft tissue infection, *n* (%)	102 (3.0)	66 (3.3)	36 (2.5)	0.178
Central nervous system infection, *n* (%)	12 (0.3)	5 (0.2)	7 (0.5)	0.244
Lactate, inflammatory and coagulation markers				
Baseline lactate, mmol/L	3.50 [2.60, 5.40]	3.70 [2.70, 5.80]	3.30 [2.60, 4.85]	**<0** **.** **001**
Follow-up lactate within 0–6 h, mmol/L	3.00 [2.00, 4.80]	3.25 [2.10, 5.40]	2.70 [1.90, 4.10]	**<0** **.** **001**
Lactate clearance 0–6 h, %	17.86 [−6.60, 37.50]	14.29 [−12.32, 36.47]	21.74 [0.00, 39.17]	**<0** **.** **001**
Lactate measurement interval, min	290.00 [230.00, 347.00]	291.00 [229.00, 350.00]	289.00 [231.00, 343.00]	0.558
Baseline white blood cell count, 10^9^/L	14.70 [9.80, 20.80]	14.40 [9.22, 20.70]	15.00 [10.50, 20.90]	**0** **.** **009**
Baseline platelet count, 10^9^/L	157.00 [113.00, 217.00]	154.50 [108.00, 216.00]	159.00 [120.00, 221.00]	**0** **.** **002**
Renal function and acid-base status				
Baseline creatinine, mg/dL	1.10 [0.80, 1.80]	1.20 [0.90, 2.00]	1.00 [0.80, 1.50]	**<0** **.** **001**
Baseline blood urea nitrogen, mg/dL	21.00 [15.00, 33.00]	22.50 [15.00, 38.00]	19.00 [14.00, 28.00]	**<0** **.** **001**
Baseline pH	7.31 [7.23, 7.37]	7.30 [7.22, 7.37]	7.31 [7.25, 7.37]	**0** **.** **003**
Baseline bicarbonate, mmol/L	21.00 [17.00, 23.00]	20.00 [17.00, 23.00]	21.00 [18.00, 23.00]	**<0** **.** **001**
Hemodynamics and vasopressor-related variables				
Minimum MAP 0–6 h, mmHg	60.00 [53.00, 65.00]	58.00 [51.00, 63.00]	63.00 [57.00, 68.00]	**<0** **.** **001**
Mean MAP 0–6 h, mmHg	73.62 [68.26, 79.14]	71.50 [66.00, 76.91]	76.50 [71.64, 81.71]	**<0** **.** **001**
Maximum heart rate 0–6 h, beats/min	97.00 [87.00, 114.00]	99.00 [88.00, 116.00]	95.00 [86.00, 110.00]	**<0** **.** **001**
Maximum respiratory rate 0–6 h, breaths/min	24.00 [20.00, 28.00]	24.00 [20.00, 29.00]	24.00 [20.00, 28.00]	**<0** **.** **001**
Minimum SpO_2_ 0–6 h, %	96.00 [93.00, 98.00]	95.00 [92.00, 98.00]	96.00 [93.00, 98.00]	**<0** **.** **001**
Number of vasopressor classes 0–6 h	1.00 [1.00, 2.00]	1.00 [1.00, 2.00]	1.00 [1.00, 2.00]	**<0** **.** **001**
Norepinephrine use, *n* (%)	1952 (56.7)	1222 (60.9)	730 (50.7)	**<0** **.** **001**
Epinephrine use, *n* (%)	820 (23.8)	483 (24.1)	337 (23.4)	0.654
Dopamine use, *n* (%)	171 (5.0)	117 (5.8)	54 (3.8)	**0** **.** **006**
Phenylephrine use, *n* (%)	2026 (58.8)	1144 (57.0)	882 (61.3)	**0** **.** **012**
Vasopressin use, *n* (%)	766 (22.2)	485 (24.2)	281 (19.5)	**0** **.** **001**
Active vasopressor time 0–6 h, min	360.00 [265.00, 360.00]	360.00 [302.00, 360.00]	360.00 [213.00, 360.00]	**<0** **.** **001**
Mean norepinephrine-equivalent dose 0–6 h, μg/kg/min	0.10 [0.04, 0.24]	0.12 [0.05, 0.27]	0.08 [0.03, 0.19]	**<0** **.** **001**
Maximum norepinephrine-equivalent dose 0–6 h, μg/kg/min	0.19 [0.10, 0.40]	0.20 [0.10, 0.46]	0.15 [0.08, 0.31]	**<0** **.** **001**
Organ support and severity scores				
Mechanical ventilation, *n* (%)	2929 (85.0)	1716 (85.5)	1213 (84.3)	0.311
Renal replacement therapy, *n* (%)	106 (3.1)	74 (3.7)	32 (2.2)	**0** **.** **014**
Acute Physiology Score III	55.00 [38.00, 78.00]	61.00 [42.00, 86.00]	48.00 [34.00, 68.00]	**<0** **.** **001**
SAPS II	45.00 [36.00, 58.00]	49.00 [39.00, 62.00]	41.00 [33.00, 52.00]	**<0** **.** **001**
OASIS	37.00 [32.00, 44.00]	39.00 [33.00, 46.00]	35.00 [30.00, 41.00]	**<0** **.** **001**

Continuous variables are presented as median [interquartile range]. Categorical variables are presented as *n* (%). Baseline and early 0–6-hour characteristics were summarized using the original, non-imputed data. *P* values were calculated using Wilcoxon rank-sum tests for continuous variables and chi-square or Fisher exact tests for categorical variables. Ethnicity and ICU type were collapsed into broader categories for readability in the publication table. Infection-site variables were not mutually exclusive.

Bold values indicate statistical significance at *P* < 0.05.

The baseline characteristics of the eICU external-validation cohort are provided in Supplementary Table S3. In the eICU cohort, 305 patients did not achieve early hemodynamic stabilization and 148 patients achieved stabilization. Similar to the MIMIC-IV cohort, patients who achieved stabilization generally showed more favorable early hemodynamic profiles, higher lactate clearance, and lower severity scores. Vasopressor-use patterns differed substantially between cohorts. During T0–6 h, norepinephrine was recorded in 56.7% of MIMIC-IV patients compared with 92.7% of eICU patients, whereas phenylephrine was recorded in 58.8% and 18.1%, respectively (Table 1; Supplementary Table S3).

Detailed missingness patterns are summarized in Supplementary Tables S4 and S5, and the variable dictionary and database-specific vasopressor and NEE processing rules are provided in Supplementary Table S6. Differential leukocyte measures were not included in the candidate predictor set because their measurement formats could not be reliably harmonized across the two databases.

Lactate clearance was calculated as [(baseline lactate—follow-up lactate)/baseline lactate] × 100%. Negative clearance values were retained. Lactate measurement interval represents the actual elapsed time between the selected baseline and follow-up measurements. Lactate clearance was not normalized to a fixed 6-hour interval.

Candidate model predictors were restricted to the variables listed in Section 2.5. Variables presented in Table 1 but not listed in Section 2.5 were included for descriptive cohort characterization only and were not used as model inputs.

### 3.2 Endpoint structure, clinical relevance, and robustness analyses

The original composite endpoint was met in 1,439 patients (41.77%) in MIMIC-IV and 148 patients (32.67%) in eICU. Among individual components, mean MAP ≥65 mmHg was achieved in 2,964 patients (86.04%) in MIMIC-IV and 371 patients (81.90%) in eICU, whereas the sustained MAP criterion, defined as MAP ≥65 mmHg for at least 80% of the T6–24-hour window, was achieved in 1,987 patients (57.68%) and 254 patients (56.07%), respectively. Absence of increase in vasopressor classes was observed in 2,918 patients (84.70%) in MIMIC-IV and 350 patients (77.26%) in eICU. Absence of increase in mean norepinephrine-equivalent dose was observed in 2,481 patients (72.02%) and 227 patients (50.11%), respectively (Supplementary Table S7).

Among patients without early hemodynamic stabilization, failure to meet the sustained MAP criterion was common in both cohorts, occurring in 1,440 patients (71.78%) in MIMIC-IV and 199 patients (65.25%) in eICU. Failure of the norepinephrine-equivalent dose non-increase criterion occurred in 964 patients (48.06%) in MIMIC-IV and 226 patients (74.10%) in eICU. Multiple failed components were present in 1,037 patients without stabilization (51.69%) in MIMIC-IV and 196 patients without stabilization (64.26%) in eICU, suggesting that non-stabilization was usually not determined by a single isolated criterion (Supplementary Table S8).

Agreement among individual endpoint components was low to moderate. In MIMIC-IV, pairwise Cohen's kappa ranged from 0.084 to 0.362, and in eICU it ranged from 0.113 to 0.440. This finding suggests that the four components captured related but non-redundant domains of early hemodynamic stabilization and vasopressor trajectory (Supplementary Table S9).

The composite endpoint showed clinical relevance. In MIMIC-IV, patients without early hemodynamic stabilization had higher ICU mortality than those with stabilization (31.16% vs. 12.65%) and higher hospital mortality (35.69% vs. 15.50%). Similar patterns were observed in eICU, where ICU mortality was 44.92% in patients without stabilization vs. 22.30% in those with stabilization, and hospital mortality was 53.77% vs. 27.03%, respectively. Mortality differences were statistically significant in both cohorts. Length-of-stay differences were less consistent, likely reflecting competing effects of early death, survival, and discharge patterns (Supplementary Table S10).

Sensitivity analyses using alternative endpoint definitions were performed using the same MIMIC-IV development framework as the primary analysis, with each alternative endpoint evaluated in its corresponding held-out 30% test subset. The rebuilt GBM models achieved AUCs of 0.734 (95% CI, 0.703–0.765) for MAP control alone, 0.745 (95% CI, 0.703–0.787) for no increase in vasopressor classes alone, 0.699 (95% CI, 0.664–0.734) for no increase in mean NEE alone, and 0.694 (95% CI, 0.661–0.727) for a ≥20% reduction in NEE. Corresponding Brier scores were 0.204, 0.113, 0.194, and 0.215, respectively. These analyses indicated that model discrimination remained broadly similar across alternative operational definitions of early stabilization. The eICU cohort was reserved for external validation of the prespecified primary composite endpoint. These analyses indicated that model discrimination remained broadly similar across alternative operational definitions of early stabilization. The eICU cohort was reserved for external validation of the prespecified primary composite endpoint (Supplementary Table S11).

To evaluate potential bias from unequal MAP measurement frequency, we repeated endpoint classification using time-standardized hourly-binned MAP values. MAP coverage was high in both cohorts. The median number of observed hourly bins was 18 (IQR, 17–18) in MIMIC-IV and 18 (IQR, 18–18) in eICU; 94.88% and 92.72% of patients with hourly MAP data, respectively, had at least 12 observed hourly intervals. Using the observed-hour denominator, agreement with the primary composite endpoint was 96.11% in MIMIC-IV (Cohen's *κ* = 0.920; 3.89% reclassified) and 97.35% in eICU (*κ* = 0.941; 2.65% reclassified). Using the more conservative fixed 18-h denominator, agreement remained high in MIMIC-IV (94.34%; *κ* = 0.883; 5.66% reclassified) and eICU (96.69%; *κ* = 0.925; 3.31% reclassified). These findings indicate that classification of early hemodynamic stabilization was highly robust to time standardization of MAP measurements (Supplementary Table S12).

Early competing events after vasopressor initiation were also summarized to characterize potential survivorship and outcome-ascertainment selection around the landmark. In a separate pre-landmark audit of 5,755 MIMIC-IV patients meeting the key baseline clinical eligibility criteria before application of the 6-hour landmark requirement, 69 patients (1.20%) died between vasopressor initiation and T6 and 6 patients (0.10%) left the ICU alive before T6. Five additional records (0.09%) had temporally inconsistent death timestamps preceding vasopressor initiation and were not eligible for landmark assessment. In eICU, 127 of 6,853 patients in the corresponding pre-landmark candidate cohort died before T6; among these, 41 met the key baseline eligibility criteria of age ≥18 years, septic shock, and baseline lactate >2 mmol/L. Patients who died before T6 could not contribute to prediction at the 6-hour landmark. During the subsequent T6–24-hour outcome window, 217 patients (6.30%) in MIMIC-IV and 48 patients (10.60%) in eICU died and were retained as non-stabilized according to the prespecified endpoint definition. ICU discharge before T0+24 h occurred in 325 patients (9.43%) in MIMIC-IV and 57 patients (12.58%) in eICU, while hospital discharge before T0+24 h occurred in 228 patients (6.62%) and 50 patients (11.04%), respectively. In MIMIC-IV, documented vasopressor discontinuation before T0+24 h was observed in 1,776 patients (51.55%). Vasopressor discontinuation was not reliably assessed in eICU because infusion records did not provide directly comparable continuous infusion end times (Supplementary Table S13).

### 3.3 Candidate-model comparison in the MIMIC-IV cohort

The performance of the candidate algorithms in the held-out MIMIC-IV model-comparison set is summarized in Table 2. Overall, the leading models showed moderate and highly comparable discrimination for predicting early hemodynamic stabilization. GBM achieved an AUC of 0.720 (95% CI, 0.688–0.751), compared with 0.719 (95% CI, 0.687–0.750) for logistic regression and 0.718 for random forest, elastic net, and XGBoost. The largely overlapping AUC estimates indicated that no clear discriminatory advantage of GBM over the other leading algorithms was demonstrated. The GBM achieved a PR-AUC of 0.635, compared with a no-skill PR-AUC of 0.418 based on the observed stabilization prevalence.

**Table 2:** Performance of candidate models in the held-out MIMIC-IV model-comparison set.

Model	AUC (95% CI)	PR-AUC	Brier score	Joint calibration intercept	Calibration slope	Threshold	Accuracy	Sensitivity	Specificity	*F*1 score
GBM	**0.720 (0.688–0.751)**	**0.635**	**0.209**	0.014	0.965	0.360	0.646	**0.794**	0.541	**0.652**
Logistic regression	0.719 (0.687–0.750)	0.619	0.210	**0.004**	0.888	0.380	0.641	0.735	0.574	0.631
Random forest	0.718 (0.687–0.750)	0.628	0.210	0.046	1.153	0.384	0.644	0.770	0.554	0.644
Elastic net	0.718 (0.687–0.750)	0.617	0.212	0.053	1.054	0.402	0.650	0.712	0.606	0.630
XGBoost	0.718 (0.686–0.749)	0.626	0.210	0.022	**0.970**	0.427	**0.654**	0.694	**0.626**	0.626
SVM, radial kernel	0.714 (0.683–0.746)	0.626	0.211	0.020	1.039	0.341	0.626	0.766	0.526	0.631
K-nearest neighbors	0.628 (0.594–0.662)	0.535	0.232	−0.135	0.778	0.445	0.585	0.568	0.597	0.534

Values are shown to three decimal places. AUC is reported with 95% confidence intervals. The prevalence of early hemodynamic stabilization in the held-out MIMIC-IV model-comparison set was 41.8%; therefore, the corresponding no-skill PR-AUC was approximately 0.418. Best values are shown in bold. For calibration, the best intercept was defined as closest to 0 and the best slope as closest to 1. AUC, area under the receiver operating characteristic curve; PR-AUC, area under the precision–recall curve; CI, confidence interval; GBM, gradient boosting machine; KNN, k-nearest neighbors; SVM, support vector machine. Classification thresholds were derived exclusively from cross-validated predictions in the MIMIC-IV training set.

The prevalence of early hemodynamic stabilization in the held-out MIMIC-IV model-comparison set was 41.8%, corresponding to a prevalence-only null-model Brier score of approximately 0.243. The GBM achieved a Brier score of 0.209, indicating lower prediction error than the null model. The joint calibration intercept reported in Table 2 was 0.014, while the calibration-in-the-large was 0.024 (95% CI, −0.114 to 0.153) and the calibration slope was 0.965 (95% CI, 0.806–1.144), indicating no substantial systematic miscalibration in the held-out MIMIC-IV model-comparison set (Supplementary Table S2). At the prespecified training-derived threshold, GBM showed relatively high sensitivity and the highest *F*1 score among the candidate models. Given the broadly comparable performance of the leading algorithms, GBM was carried forward as a representative nonlinear model for subsequent external validation and interpretability analyses rather than as evidence of superiority over the other leading approaches.

The ROC curves of the candidate models are shown in Figure 2a and were largely overlapping among the leading algorithms. Decision-curve analysis is shown in Figure 2b; GBM showed positive net benefit across the examined threshold range, with substantial overlap among the leading models. Calibration of the GBM model is shown in Figure 2c, demonstrating reasonable agreement between predicted and observed probabilities.

**Figure 2:**
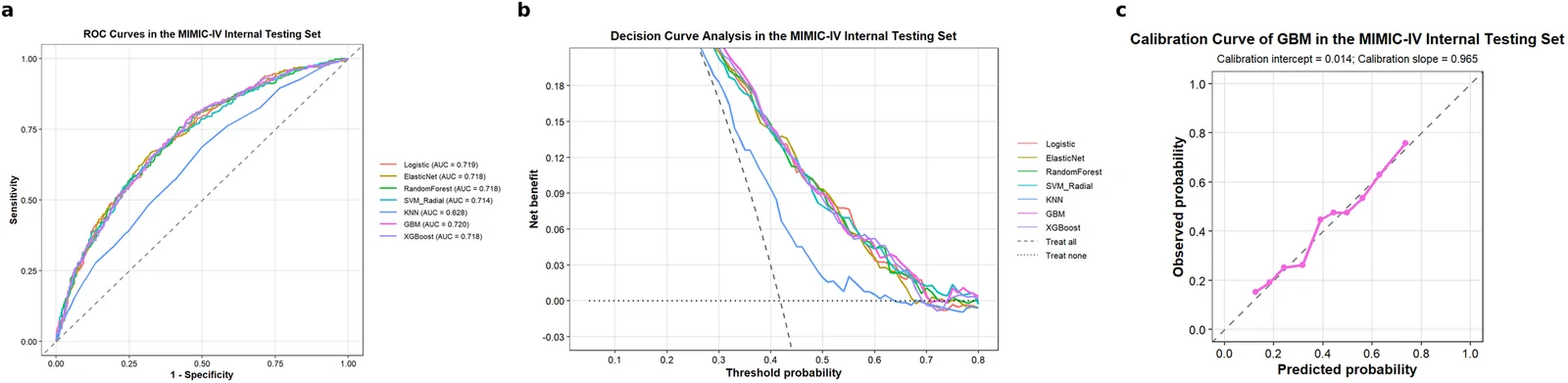
Performance of candidate models in the held-out MIMIC-IV model-comparison set. **(a)** Receiver operating characteristic curves of candidate models. **(b)** Decision curve analysis of candidate models. **(c)** Calibration plot of the primary GBM model. AUC, area under the receiver operating characteristic curve; GBM, gradient boosting machine.

The calibration intercept shown in this table was estimated jointly with the calibration slope. For the primary GBM, calibration-in-the-large and bootstrap 95% confidence intervals for calibration metrics are reported separately in Supplementary Table S2.

### 3.4 External validation in the eICU cohort

The external-validation performance of the candidate models in the eICU cohort is summarized in Table 3. The primary GBM achieved an ROC-AUC of 0.730 (95% CI, 0.683–0.778), a PR-AUC of 0.546, and a Brier score of 0.192. XGBoost showed similar discrimination (ROC-AUC, 0.728; 95% CI, 0.680–0.776), whereas the remaining candidate models showed lower discrimination. At the classification threshold of 0.360 derived exclusively from cross-validated predictions in the MIMIC-IV training set, the GBM had an accuracy of 0.689, sensitivity of 0.466, specificity of 0.797, and *F*1 score of 0.495. Because failure to achieve stabilization may represent the more clinically actionable state, the same locked rule was also expressed as non-stabilization risk without threshold re-optimization in eICU; the corresponding sensitivity and specificity for identifying non-stabilization were 0.797 and 0.466, respectively.

**Table 3:** External validation performance of candidate models in the eICU cohort.

Model	Threshold	AUC (95% CI)	PR-AUC	Accuracy	Sensitivity	Specificity	Precision	NPV	*F*1 score	Brier score	Calibration-in-the-large	Calibration slope
GBM	0.360	0.730 (0.683–0.778)	0.546	0.689	0.466	0.797	0.527	0.755	0.495	0.192	0.234	1.079
XGBoost	0.427	0.728 (0.680–0.776)	0.557	0.691	0.338	0.862	0.543	0.729	0.417	0.190	0.185	1.055
Elastic net	0.402	0.696 (0.644–0.749)	0.512	0.700	0.372	0.859	0.561	0.738	0.447	0.201	0.163	0.326
Random forest	0.384	0.685 (0.633–0.736)	0.513	0.680	0.426	0.803	0.512	0.742	0.465	0.199	0.069	1.139
Logistic regression	0.380	0.689 (0.636–0.742)	0.507	0.698	0.439	0.823	0.546	0.751	0.487	0.204	0.233	0.232
SVM, radial kernel	0.341	0.674 (0.622–0.726)	0.495	0.675	0.399	0.810	0.504	0.735	0.445	0.203	0.045	1.262
K-nearest neighbors	0.445	0.614 (0.560–0.669)	0.428	0.623	0.412	0.725	0.421	0.718	0.416	0.217	−0.304	0.754

AUC, area under the receiver operating characteristic curve; PR-AUC, area under the precision–recall curve; NPV, negative predictive value. The prevalence of early hemodynamic stabilization in the eICU cohort was 32.67%; therefore, the corresponding no-skill PR-AUC was approximately 0.327. A calibration-in-the-large value of 0 and a calibration slope of 1 indicate ideal calibration. Classification thresholds were derived exclusively from cross-validated predictions in the MIMIC-IV training set and applied unchanged to the eICU cohort. Detailed calibration estimates with bootstrap 95% confidence intervals are provided in Supplementary Table S16.

External calibration showed modest systematic drift. The observed stabilization prevalence was 32.7%, compared with a mean predicted probability of 28.3%, corresponding to a calibration-in-the-large of 0.234 (95% CI, 0.023–0.429). The calibration slope remained close to unity at 1.079 (95% CI, 0.815–1.357), while the integrated calibration index was 0.053 (95% CI, 0.025–0.094). Calibration transportability varied across algorithms, with more attenuated slopes observed for logistic regression and elastic net (Table 3). The external ROC, decision, and calibration curves are shown in Figure 3. Decision-curve analysis, expressed using failure to achieve stabilization as the actionable event, compared the GBM with full logistic regression and the parsimonious MAP–NEE model; detailed net-benefit estimates and paired comparisons are reported in Supplementary Table S14. Detailed calibration estimates are provided in Supplementary Table S16.

**Figure 3:**
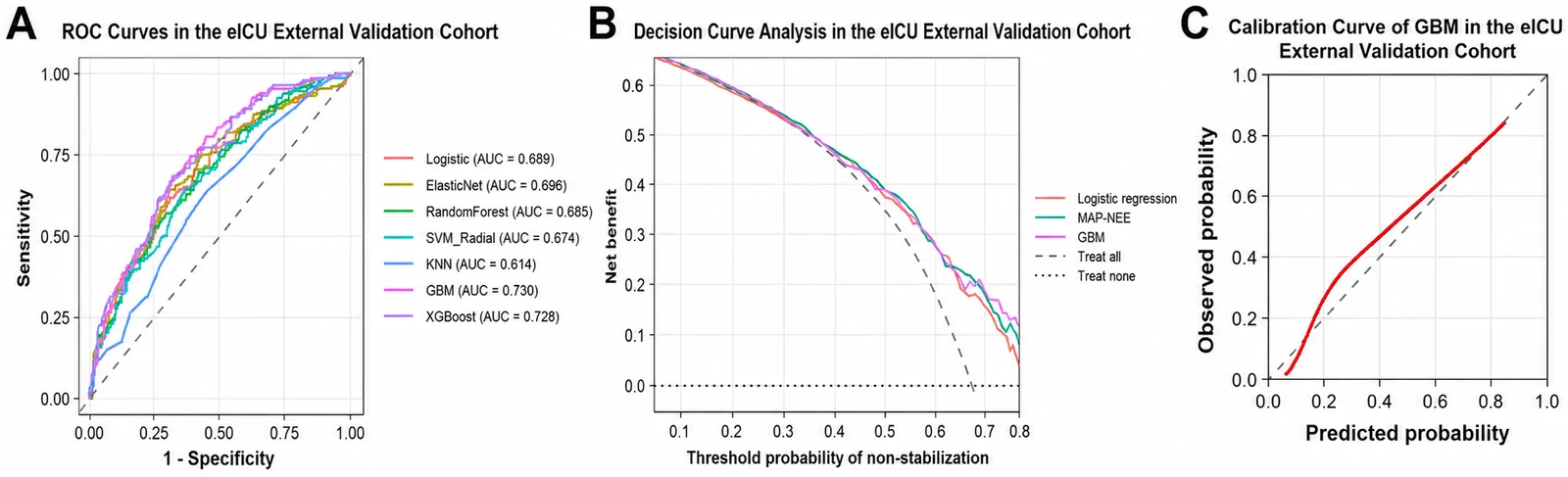
External validation performance of candidate models in the eICU cohort. **(A)** Receiver operating characteristic curves of candidate models in the eICU external validation cohort. **(B)** Decision-curve analysis using failure to achieve early hemodynamic stabilization as the clinically actionable event, comparing the primary GBM model with full logistic regression and the parsimonious MAP–NEE benchmark model. Treat-all and treat-none strategies are shown for reference. **(C)** Flexible calibration curve of the primary GBM model in the eICU external-validation cohort. The diagonal dashed line represents perfect calibration.

In a *post-hoc* secondary analysis, logistic recalibration aligned the mean predicted probability with the observed stabilization prevalence and modestly reduced the Brier score from 0.192 to 0.190 and the integrated calibration index from 0.053 to 0.028. Because recalibration was developed and evaluated within the same eICU cohort, these findings were considered exploratory and were not interpreted as independent validation of the recalibrated model (Supplementary Table S16).

The 453 patients in the eICU cohort were distributed across 47 hospitals, with a median of 3 eligible patients per hospital (IQR, 1–12); only six hospitals met the prespecified minimum sample-size criteria for exploratory site-specific evaluation. Across these hospitals, AUC estimates ranged from 0.639 to 0.713, although precision was limited by small sample sizes. Exploratory subgroup analyses across age, sex, ICU type, mechanical ventilation, infection source, and baseline lactate generally showed moderate but heterogeneous discrimination, with less precise estimates in smaller subgroups. Notably, the surgical/trauma ICU subgroup included only 35 patients and had an AUC of 0.434 (95% CI, 0.227–0.640). Accordingly, site- and subgroup-specific findings were considered exploratory and descriptive rather than evidence of differential model performance (Supplementary Table S15).

The observed stabilization prevalence was 32.67%, corresponding to a prevalence-only null-model Brier score of approximately 0.220.

CITL, calibration-in-the-large; ICI, integrated calibration index; NPV, negative predictive value. A CITL of 0 and a calibration slope of 1 indicate ideal calibration. Classification thresholds were derived exclusively from cross-validated predictions in the MIMIC-IV training set and applied unchanged to the eICU cohort. Detailed calibration estimates with bootstrap 95% confidence intervals are provided in Supplementary Table S16.

### 3.5 Comparison with simple hemodynamic benchmarks and reduced model analysis

To assess the incremental predictive value of the full GBM beyond simple early hemodynamic information, we compared it with several clinical benchmark models (Supplementary Table S17). In the held-out MIMIC-IV model-comparison set, the full GBM achieved an AUC of 0.720 (95% CI, 0.688–0.751), compared with 0.675 (95% CI, 0.642–0.707) for mean MAP alone and 0.687 (95% CI, 0.655–0.720) for the parsimonious MAP–NEE model. The corresponding differences in AUC were 0.045 (95% CI, 0.024–0.065; *P* < 0.001) and 0.032 (95% CI, 0.015–0.049; *P* < 0.001), respectively.

In the eICU external validation cohort, however, the incremental advantage of the full GBM over simple hemodynamic models was attenuated. Mean MAP alone achieved an AUC of 0.735 (95% CI, 0.688–0.782), compared with 0.730 (95% CI, 0.683–0.778) for the full GBM (ΔAUC, −0.005; 95% CI, −0.039 to 0.030; *P* = 0.789). Similarly, the parsimonious MAP–NEE model achieved an AUC of 0.708 (95% CI, 0.657–0.759), with no significant difference from the full GBM (ΔAUC, 0.022; 95% CI, −0.015 to 0.060; *P* = 0.238).

Comparison with conventional logistic regression showed no evidence of incremental discrimination for the full GBM in the held-out MIMIC-IV model-comparison set (AUC, 0.720 vs. 0.719; ΔAUC, 0.001; 95% CI, −0.015 to 0.016; *P* = 0.902). Overall prediction error was also nearly identical, with Brier scores of 0.209 for GBM and 0.210 for logistic regression (ΔBrier, 0.002; 95% CI, −0.004 to 0.007). Calibration was broadly comparable in the MIMIC-IV cohort, with calibration intercepts of 0.014 and 0.004 and calibration slopes of 0.965 and 0.888 for GBM and logistic regression, respectively. In the eICU external validation cohort, however, the incremental advantage of the full GBM was attenuated. The full GBM showed higher discrimination than the original logistic regression model (AUC, 0.730 vs. 0.689; ΔAUC, 0.041; 95% CI, 0.005 to 0.080; *P* = 0.042) and a lower Brier score (0.192 vs. 0.204; ΔBrier, 0.012; 95% CI, 0.001 to 0.024; *P* = 0.041). Detailed external calibration results for the full GBM and logistic regression are reported in Section 3.4 and Supplementary Table S16. After exclusion of the five proximal hemodynamic and vasopressor-related predictors, discrimination decreased substantially.

The reduced GBM achieved an AUC of 0.662 (95% CI, 0.628–0.695) in the held-out MIMIC-IV model-comparison set, compared with 0.720 for the full GBM (ΔAUC, 0.058; 95% CI, 0.030–0.086; *P* < 0.001). In the eICU cohort, discrimination decreased from an AUC of 0.730 for the full GBM to 0.630 (95% CI, 0.575–0.684) for the reduced GBM (ΔAUC, 0.101; 95% CI, 0.054–0.145; *P* < 0.001). The Brier score increased from 0.192 to 0.211 (ΔBrier, 0.019; 95% CI, 0.009–0.028; *P* = 0.001) (Figure 4; Supplementary Table S17).

**Figure 4:**
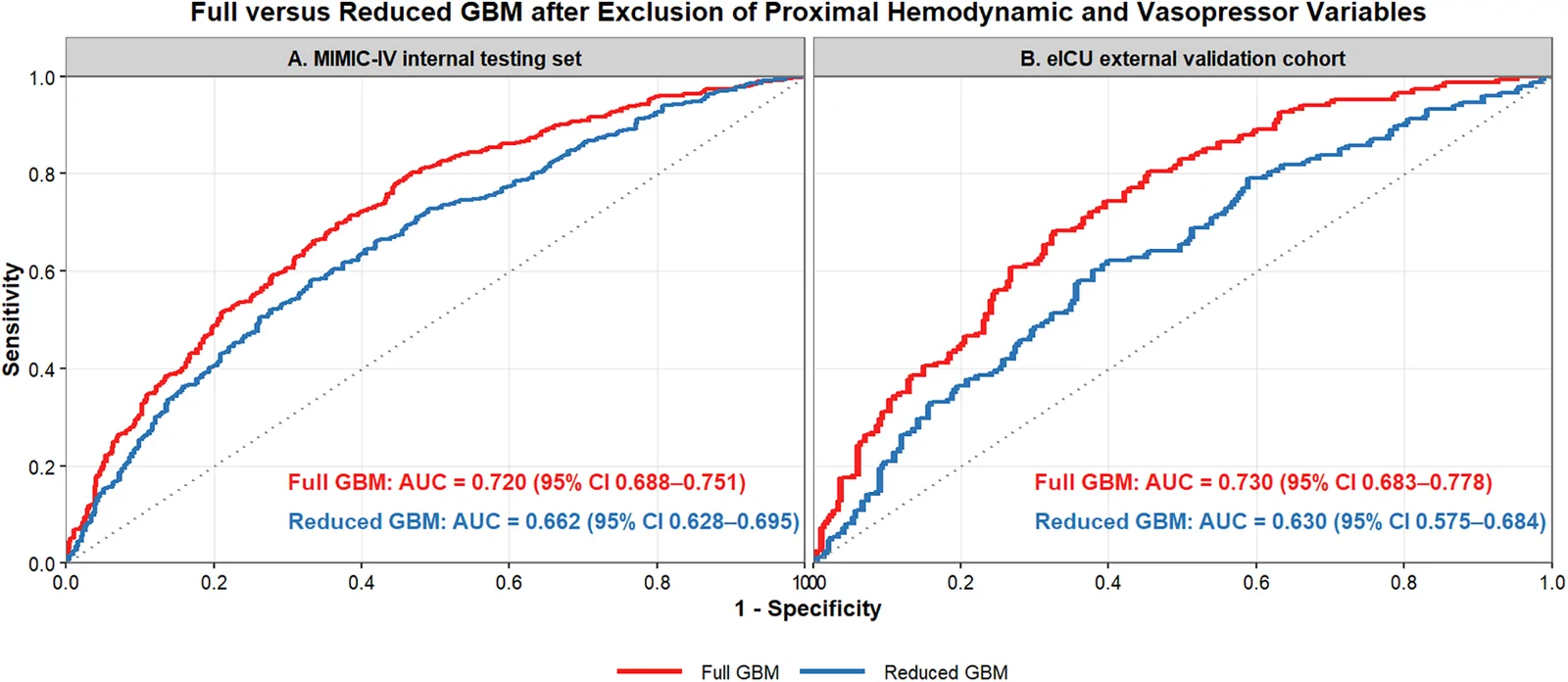
Discrimination of the full and reduced GBM models after exclusion of proximal hemodynamic and vasopressor-related predictors. **(A)** Held-out MIMIC-IV model-comparison set. **(B)** eICU external-validation cohort.

Decision-curve analysis was additionally expressed using failure to achieve early hemodynamic stabilization as the clinically actionable event. In the eICU cohort, the absolute net benefits of the full GBM were 0.593, 0.533, and 0.462 at illustrative threshold probabilities of 0.20, 0.30, and 0.40, respectively. The corresponding net benefits were 0.584, 0.529, and 0.467 for full logistic regression and 0.590, 0.536, and 0.467 for the parsimonious MAP–NEE model. At a threshold of 0.20, the GBM showed a small increase in net benefit relative to logistic regression (ΔNB, 0.008; 95% CI, 0.001–0.018), whereas the difference relative to the MAP–NEE model was small and its confidence interval included zero (ΔNB, 0.003; 95% CI, −0.001 to 0.008). At thresholds of 0.30 and 0.40, differences between the GBM and both comparator models were small, with confidence intervals including or reaching zero (Figure 3B; Supplementary Table S14). Overall, the decision-curve analysis did not demonstrate a consistent incremental net benefit of the GBM over simpler regression-based approaches in the external-validation cohort.

These findings indicate that proximal hemodynamic information, particularly early MAP trajectories, accounted for a substantial, but not exclusive, proportion of predictive performance. Vasopressor-intensity variables also contributed to model prediction, although their exclusion alone did not materially reduce discrimination. Although the full GBM provided modest incremental discrimination over simple hemodynamic benchmarks in internal validation, this advantage was not consistently maintained during external validation.

### 3.6 Model interpretability

Model interpretability was assessed using SHAP analysis for the primary GBM model. The SHAP summary plot is shown in Figure 5. The most influential predictors included mean MAP during the first 6 h, minimum MAP during the first 6 h, age, baseline platelet count, lactate clearance during the first 6 h, baseline white blood cell count, baseline blood urea nitrogen, baseline creatinine, maximum norepinephrine-equivalent dose, and admission weight. Overall, hemodynamic variables, lactate dynamics, inflammatory and coagulation markers, renal function indicators, and treatment intensity contributed substantially to model prediction.

**Figure 5:**
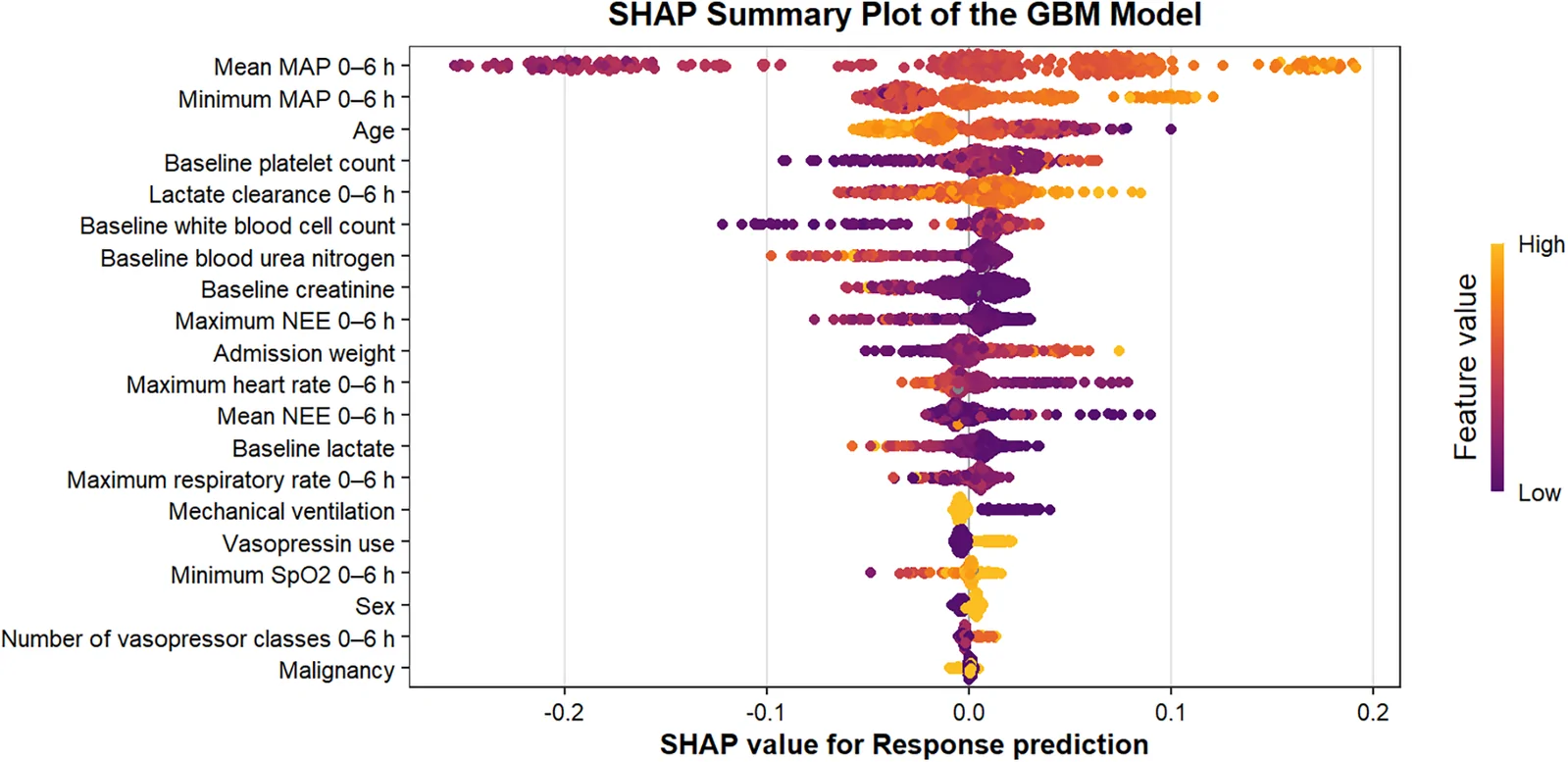
SHAP summary plot of the primary GBM model.

Positive SHAP values indicated a higher predicted probability of early hemodynamic stabilization. Higher mean and minimum MAP values during the first 6 h were generally associated with a higher predicted probability of early hemodynamic stabilization, whereas lower MAP values contributed negatively to stabilization prediction. Higher lactate clearance and higher platelet count tended to increase the predicted probability of early hemodynamic stabilization. In contrast, worse renal function, reflected by higher blood urea nitrogen and creatinine levels, and greater vasopressor intensity, reflected by higher norepinephrine-equivalent doses, were generally associated with a lower predicted probability of early hemodynamic stabilization.

Variable importance analysis showed a similar ranking pattern, with mean MAP during the first 6 h being the dominant predictor, followed by minimum MAP, lactate clearance, platelet count, mean norepinephrine-equivalent dose, and white blood cell count (Supplementary Figure S1). SHAP dependence plots further demonstrated nonlinear associations between key predictors and model output, particularly for MAP, lactate clearance, platelet count, and white blood cell count (Supplementary Figure S2). Partial dependence plots showed broadly consistent patterns and are provided in Supplementary Figure S3. The SHAP dependence and partial-dependence displays showed broadly consistent descriptive patterns for several key predictors, including MAP, lactate clearance, platelet count, and white blood cell count. These findings were interpreted as patterns in model attribution and model output rather than as evidence of causal, treatment-related, or physiological mechanisms.

### 3.7 Sensitivity analyses

Sensitivity analyses generally supported the robustness of the primary findings. Excluding mean and maximum norepinephrine-equivalent dose (NEE), with or without additional exclusion of vasopressor-class count, did not materially reduce discrimination. The corresponding AUCs were 0.722 and 0.723 in the MIMIC-IV held-out sets and 0.739 (95% CI, 0.692–0.785) for both specifications in the eICU cohort, compared with 0.730 for the full GBM; neither reduced specification differed significantly from the full model in eICU (paired DeLong *P* = 0.245 and *P* = 0.241, respectively) (Supplementary Table S18).

Additional analyses addressing outcome classification and cohort selection yielded broadly consistent results. All 217 deaths during the T6–24-h outcome window were already classified as non-stabilization, and explicitly enforcing this rule caused no reclassification. In contrast, treating successful vasopressor discontinuation before T0+24 h as stabilization reclassified 607 patients (17.62%) and increased the stabilization prevalence from 41.77% to 59.39%; nevertheless, discrimination remained preserved after model rebuilding (AUC, 0.766; 95% CI, 0.737–0.795). Relaxing the requirement for complete baseline white blood cell and platelet measurements increased the cohort from 3,445 to 3,529 patients and yielded similar performance (AUC, 0.714; 95% CI, 0.683–0.745). Explicitly incorporating predictor-missingness indicators likewise produced essentially unchanged discrimination (AUC, 0.720; 95% CI, 0.688–0.751) (Supplementary Table S19).

Finally, serial lactate availability was examined as a potential source of care-intensity-related selection. Among 5,338 otherwise eligible patients, 1,893 (35.46%) lacked an eligible repeat lactate measurement. After removing the repeat-lactate requirement and lactate clearance from the predictor set, the baseline-lactate-only GBM retained moderate discrimination (AUC, 0.698; 95% CI, 0.673–0.723) with acceptable calibration (intercept, 0.008; slope, 0.932) (Supplementary Table S20). Overall, predictive performance was broadly preserved across these sensitivity analyses, although the substantial reclassification associated with successful early vasopressor discontinuation underscores that the absolute definition of early hemodynamic stabilization remains sensitive to its operational specification.

## 4 Discussion

In this multicohort study, we developed, internally validated, externally validated, and interpreted machine learning models for predicting early hemodynamic stabilization after vasopressor initiation in patients with septic shock using routinely available early dynamic clinical features. The GBM model showed moderate discrimination in the held-out MIMIC-IV model-comparison set and retained similar discrimination in the independent eICU external-validation cohort, although modest systematic calibration drift was observed externally. Additional endpoint analyses showed that the operational composite endpoint was associated with ICU and hospital mortality and was robust to hourly-binned MAP definitions. However, comparison with simple clinical benchmark and reduced models indicated that proximal hemodynamic information, particularly early MAP trajectories, accounted for a substantial proportion of model performance. These findings suggest that the model should be interpreted as a dynamic risk-stratification tool for early hemodynamic stabilization rather than as a direct measure of pharmacological vasopressor responsiveness.

Septic shock is characterized by profound circulatory, cellular, and metabolic abnormalities and is associated with high mortality despite advances in early recognition and resuscitation (1, 2). Current guidelines recommend norepinephrine as the first-line vasopressor and commonly use an initial MAP target of approximately 65 mmHg during early resuscitation (2, 3). However, the clinical response to vasopressors varies substantially among patients. Some patients rapidly achieve sustained arterial pressure control without requiring escalation of vasoactive support, whereas others remain vasopressor-dependent despite apparently similar initial treatment. This heterogeneity highlights the need for tools that can summarize early treatment response and identify patients at risk of persistent hemodynamic instability.

A key feature of this study is the use of a 6-hour landmark after vasopressor initiation. This time point was selected because the first several hours after recognition and treatment of septic shock represent a clinically important period for early resuscitation, reassessment, and treatment adjustment. The concept of a 6-hour early resuscitation window has long been embedded in septic shock research, including early goal-directed therapy, in which protocolized hemodynamic resuscitation was implemented during the first 6 h after presentation (17). Early lactate clearance has also been associated with improved outcomes in severe sepsis and septic shock and has been evaluated as an early resuscitation target within a similar early treatment window (4, 18). Although current guidelines emphasize earlier recognition and intervention, they also recommend repeated reassessment of perfusion and serial lactate measurement during resuscitation (2, 19–21). Therefore, the 6-hour landmark in the present study was intended to reflect a practical point at which sufficient early treatment information has accumulated while prediction may still be early enough to support reassessment before subsequent persistent hemodynamic instability or treatment escalation.

Another important feature of our study is the use of early dynamic clinical features rather than isolated baseline measurements. Early hemodynamic stabilization is not a static phenomenon. It evolves with changes in arterial pressure, lactate clearance, vasopressor dose requirement, respiratory status, renal function, and organ support during early shock management. Prior machine learning studies in sepsis have increasingly highlighted the value of electronic health record data and dynamic clinical patterns for early prediction (22–25). In the present study, features extracted during the T0–6-hour window, such as mean and minimum MAP, lactate clearance, mean and maximum norepinephrine-equivalent dose, vital-sign extremes, and organ support, were designed to summarize early physiologic evolution after vasopressor initiation. This approach differs from models that rely mainly on single baseline or pre-treatment values and may better reflect the trajectory of early shock reversal.

Previous studies have examined vasopressor responsiveness from different perspectives. Scheibner et al. developed and externally validated a machine learning model to predict adjunctive vasopressin responsiveness among patients with septic shock treated with norepinephrine and vasopressin (6). That study provided important evidence that machine learning could help characterize response to a specific second-line vasopressor, but the outcome focused on vasopressin responsiveness and was mainly defined by norepinephrine dose reduction after vasopressin initiation. More recently, Chen et al. proposed the blood pressure response index as a simplified measure of cardiovascular response to vasoactive drugs and demonstrated its association with clinical outcomes across multicenter sepsis cohorts (7). Our study extends these prior efforts in several ways. First, rather than focusing on response to a specific vasopressor, we evaluated an operational early hemodynamic stabilization phenotype based on sustained MAP control and absence of vasopressor escalation. Second, we used a landmark framework in which early dynamic features were extracted during the first 6 h after vasopressor initiation and early hemodynamic stabilization was assessed during the subsequent 6–24-hour window. Third, the model integrated multiple clinically interpretable domains, including MAP stability, lactate dynamics, vasopressor intensity, vital signs, renal function, inflammatory and coagulation markers, and organ support, and was externally validated in an independent multicenter cohort.

Because no universally accepted gold standard exists for vasopressor responsiveness in retrospective ICU databases, the primary endpoint should be interpreted as an operational measure of early hemodynamic stabilization rather than a direct biological measure of pharmacological vasopressor responsiveness. Sensitivity analyses provided a more nuanced assessment of the stability of this operational endpoint. Explicit classification of deaths during the outcome window as non-stabilization did not alter any outcome assignments, indicating that death was already appropriately captured by the primary composite endpoint. In contrast, treating successful early vasopressor discontinuation as stabilization reclassified 607 patients (17.62%), increasing the stabilization prevalence from 41.77% to 59.39%. This finding indicates that the absolute classification of early stabilization was sensitive to how successful early vasopressor withdrawal was operationalized. Importantly, predictive discrimination remained preserved after rebuilding the GBM using this alternative endpoint definition, with an AUC of 0.766. Taken together, these findings suggest that although endpoint classification depends to some extent on its operational definition, the underlying predictive signal remained broadly stable across clinically plausible alternative specifications. Similarly, relaxing the baseline WBC and platelet completeness requirements increased the cohort to 3,529 patients while yielding performance comparable to the primary model, reducing concern that the main findings were driven by complete-case cohort selection. The additional endpoint analyses support this interpretation. Individual components showed low-to-moderate agreement, indicating that the composite endpoint captured complementary domains rather than redundant measurements. Moreover, patients without early hemodynamic stabilization had substantially higher ICU and hospital mortality in both MIMIC-IV and eICU, supporting the clinical relevance of the operational endpoint.

The moderate discrimination observed in this study should be interpreted in the clinical context of early septic shock reassessment. Early hemodynamic stabilization after vasopressor initiation is not determined by a single physiologic variable but reflects the interaction of vascular tone, cardiac function, fluid responsiveness, inflammatory state, metabolic recovery, organ dysfunction, clinician titration decisions, and treatment intensity (26, 27). Therefore, an AUC in the moderate range may still provide clinically meaningful risk stratification, particularly when the model is intended to support early reassessment rather than replace bedside judgment. The comparable performance of GBM and XGBoost also suggests that the predictive signal was not specific to one modeling algorithm. Given the broadly comparable performance of the leading algorithms, GBM was carried forward as a representative nonlinear model because it offered competitive overall predictive performance together with straightforward model-interpretation analyses; this choice should not be interpreted as evidence of superiority over the other leading algorithms.

External validation also revealed an important distinction between discrimination and calibration transportability. Although the GBM calibration slope remained close to unity at 1.079 (95% CI, 0.815–1.357), the positive calibration-in-the-large of 0.234 (95% CI, 0.023–0.429) and the lower mean predicted probability relative to the observed stabilization prevalence (28.3% vs. 32.7%) indicated modest systematic underprediction in the eICU cohort. This calibration drift may reflect differences in case mix, treatment patterns, outcome prevalence, and data-generating processes between MIMIC-IV and eICU (28). In the *post-hoc* secondary recalibration analysis, logistic recalibration reduced the ICI from 0.053 to 0.028 and modestly improved the Brier score from 0.192 to 0.190, suggesting that local recalibration may improve absolute probability estimates. However, because recalibration was developed and evaluated within the same eICU cohort, these findings should be considered exploratory and require evaluation in an independent contemporary cohort before clinical implementation.

SHAP analysis was used to characterize the contribution of individual predictors to the GBM predictions rather than to identify physiological mechanisms. Mean and minimum MAP during the first 6 h showed the largest model attributions, indicating that the model relied strongly on the early hemodynamic trajectory. Lactate clearance, platelet count, white blood cell count, creatinine, blood urea nitrogen, and norepinephrine-equivalent dose also contributed to model predictions. The directions of several of these attribution patterns were consistent with established clinical associations in septic shock; however, such consistency should not be interpreted as validation of biological mechanisms or causal effects. In particular, early MAP and vasopressor-intensity measures are physiologically and clinically related, and correlated predictors may share or redistribute SHAP attribution. Therefore, the relative ranking of individual predictors should not be interpreted as their independent importance. Formal resampling-based uncertainty or stability intervals for SHAP feature rankings were not estimated in this study. The broadly concordant patterns observed across SHAP, conventional variable-importance, and partial-dependence displays provide complementary descriptive information about model behavior but do not substitute for formal attribution-stability analysis.

The additional sensitivity analyses are important for interpreting the credibility and limitations of this prediction task. Alternative endpoint analyses in held-out MIMIC-IV test subsets showed broadly similar discrimination across operational definitions, with ROC-AUCs ranging from 0.694 to 0.745. These findings indicate that predictive discrimination was broadly preserved across reasonable alternative endpoint definitions, while also emphasizing that the absolute classification of early stabilization depended on its specific operationalization. Because these analyses were intended as endpoint-definition sensitivity analyses rather than separate externally validated prediction tasks, the eICU cohort was retained for external validation of the prespecified primary composite endpoint. This finding supports interpreting the model as predicting early hemodynamic stabilization rather than a generalizable measure of true pharmacological vasopressor responsiveness. Because the endpoint incorporated MAP control and absence of vasopressor escalation, potential circularity between predictors and outcome definition was a legitimate concern. The comparison with simple clinical baseline models showed that the full GBM model did not clearly outperform simple MAP-based or MAP-plus-norepinephrine-equivalent-dose models in external validation. Consistent with these discrimination analyses, decision-curve comparisons showed that the incremental net benefit of the full GBM over simpler regression-based models was modest, particularly in the external validation cohort. Moreover, removal of direct MAP and norepinephrine-equivalent-dose predictors substantially reduced model performance. These findings indicate that the model primarily captured continuity of early hemodynamic status and vasopressor-dose trajectory. Therefore, the value of the model lies more in structured early risk stratification using a landmark dynamic prediction framework than in demonstrating clear superiority of machine learning over simple hemodynamic predictors. The hourly-binned MAP sensitivity analysis further suggests that endpoint classification was robust to differences in MAP measurement frequency. Although raw MAP recording frequency differed between databases, the use of hourly-binned median MAP values produced classifications that were highly consistent with the original endpoint and preserved the association with lower mortality. This finding reduces concern that the sustained MAP component was driven primarily by unequal measurement density.

Serial lactate testing represented an additional potential source of care-intensity-related selection because more than one-third of otherwise eligible patients did not undergo repeat lactate measurement during the early predictor window. Repeat-lactate availability was statistically associated with subsequent stabilization status, although the effect size was small. Importantly, expanding the cohort to patients without repeat lactate measurements and removing lactate clearance from the predictor set resulted in only a modest reduction in discrimination, from an AUC of 0.720 in the primary model to 0.698 in the baseline-lactate-only sensitivity model, while calibration remained acceptable. These findings reduce, although do not eliminate, concern that the primary results were predominantly driven by selection related to serial lactate monitoring.

The potential clinical value of this model lies in early risk stratification rather than direct treatment prescription. At the 6-hour landmark after vasopressor initiation, the model may provide probabilistic risk stratification for subsequent failure to achieve sustained hemodynamic stabilization during the T6–24-hour period. Such risk estimates could potentially support closer hemodynamic reassessment, including evaluation of fluid responsiveness, infection source control, cardiac function, vasopressor requirements, and other potentially reversible contributors to persistent shock, rather than directly determining treatment escalation. In a potential future reassessment workflow, a false-positive high-risk prediction might lead primarily to additional clinical reassessment in a patient who subsequently stabilizes, whereas a false-negative prediction could theoretically contribute to delayed recognition of persistent hemodynamic instability. These potential consequences remain hypothetical because the model has not yet undergone prospective clinical impact evaluation. Although the originally reported sensitivity for identifying subsequent stabilization was only 46.6%, expressing the same locked classification rule for the clinically actionable complementary outcome of non-stabilization yielded a sensitivity of approximately 79.7%. Nevertheless, decision-curve analysis did not demonstrate a consistent incremental net benefit of the GBM over the full logistic regression or parsimonious MAP–NEE models during external validation. Accordingly, the model should be viewed as a probabilistic risk-stratification and reassessment tool requiring prospective evaluation rather than as a replacement for clinical judgment or as a direct basis for vasopressor selection, dose adjustment, or treatment escalation.

This study has several strengths. First, we used a landmark design to separate the predictor window from the outcome assessment window, thereby reducing temporal ambiguity and information leakage. Second, the 6-hour landmark was clinically motivated by the early resuscitation period in septic shock, during which hemodynamic response, lactate kinetics, and vasopressor requirement are repeatedly reassessed (4, 17–19, 29). This choice is also consistent with randomized trials and consensus recommendations emphasizing early shock control, vasopressor strategy, and structured hemodynamic reassessment in septic shock (30–35). Third, the model was developed in MIMIC-IV and externally validated in the multicenter eICU cohort, which improves the credibility of model transportability. Fourth, the outcome definition incorporated both sustained MAP control and absence of vasopressor escalation, making it more clinically meaningful than a single blood pressure or dose threshold. Fifth, model evaluation included discrimination, calibration, Brier score, decision curve analysis, and external validation rather than relying solely on AUC (36–39). Finally, SHAP analysis and complementary variable-importance and partial-dependence displays improved transparency by providing descriptive views of how the fitted model used the available predictors.

Several limitations should be acknowledged. First, the retrospective observational design and pragmatic composite endpoint may have introduced residual confounding, measurement error, documentation error, and outcome misclassification; therefore, the identified predictor–outcome associations should not be interpreted as causal or as direct measures of pharmacological vasopressor responsiveness. Important clinical information, including detailed fluid responsiveness, echocardiographic and microcirculatory measures, clinician titration intent, cumulative fluid balance, and source-control timing, could not be consistently harmonized across both databases and was therefore not included. Second, serial lactate availability, variable MAP measurement frequency, and the use of median/mode imputation may have introduced selection and measurement biases that cannot be fully eliminated despite sensitivity analyses. Third, the model generated a single prediction at the 6-hour landmark; earlier predictions, such as at 1–3 h after vasopressor initiation, and serially updated dynamic predictions were not evaluated and should be investigated in future studies. Fourth, the external-validation cohort was modest in size and unevenly distributed across eICU hospitals. Although 453 patients were drawn from 47 hospitals, the median number of eligible patients per hospital was only 3 (IQR, 1–12), limiting reliable hospital-level internal-external validation and the precision of several site- and subgroup-specific estimates. Differences in database structure, sepsis-phenotype operationalization, measurement frequency, vasopressor-use patterns, and medication documentation between MIMIC-IV and eICU may have contributed to differences in calibration and transportability. In particular, norepinephrine and phenylephrine use differed substantially between cohorts. The higher phenylephrine exposure observed in MIMIC-IV may partly reflect differences in case mix, local prescribing practices, and database-specific medication documentation; because the indication for individual phenylephrine infusions could not be reliably reconstructed, phenylephrine exposure should not be interpreted as specific treatment for septic vasoplegia. In addition, MIMIC-IV supported elapsed-time-weighted NEE calculation from documented infusion intervals, whereas eICU NEE was summarized across recorded infusion offsets because directly comparable continuous infusion end times were unavailable. These database-specific differences should be considered when interpreting external calibration and transportability. Accordingly, subgroup and site-specific analyses should be considered exploratory, and the eICU validation primarily supports cross-database and multicenter transportability within retrospective US critical care data rather than contemporary international generalizability. Fifth, SHAP values represent model attributions rather than causal or mechanistic effects. Correlated predictors, particularly MAP and vasopressor-intensity measures, may share or redistribute attribution, and formal correlation-adjusted SHAP analyses or resampling-based uncertainty estimates for feature rankings were not performed. Finally, the study evaluated retrospective predictive performance rather than prospective clinical impact; therefore, the model should be regarded as a risk-stratification and reassessment tool rather than a basis for automatic treatment decisions. Larger contemporary multicenter and prospective studies are needed to confirm calibration, transportability, attribution stability, and clinical usefulness, and to determine whether model-guided use improves clinical workflows or patient outcomes.

## 5 Conclusion

An interpretable machine learning model based on early dynamic clinical features showed moderate discrimination in the held-out MIMIC-IV model-comparison set and similar discrimination in the independent eICU cohort for predicting early hemodynamic stabilization after vasopressor initiation in patients with septic shock. However, model performance was substantially driven by proximal hemodynamic features, particularly early MAP trajectories, and the incremental value beyond simple hemodynamic models was limited. The operational composite endpoint was associated with ICU and hospital mortality, supporting its clinical relevance while also highlighting the importance of endpoint operationalization. These findings support the potential value of structured early risk stratification after vasopressor initiation, but prospective validation and clinical workflow evaluation are required before implementation.

## Data Availability

Publicly available datasets were analyzed in this study. This data can be found here: The MIMIC-IV database and the eICU Collaborative Research Database are available from PhysioNet after completion of the required credentialing and data use agreements. The processed analytic code and derived datasets can be made available by the corresponding authors upon reasonable request, subject to the data use agreements of the original databases.
